# Depolarization-Evoked Secretion Requires Two Vicinal Transmembrane Cysteines of Syntaxin 1A

**DOI:** 10.1371/journal.pone.0001273

**Published:** 2007-12-05

**Authors:** Roy Cohen, Merav Marom, Daphne Atlas

**Affiliations:** Department of Biological Chemistry, The Alexander Silberman Institute of Life Sciences, The Hebrew University of Jerusalem, Jerusalem, Israel; Vrije Universiteit Amsterdam, Netherlands

## Abstract

**Background:**

The interactions of the voltage-gated Ca^2+^ channel (VGCC) with syntaxin 1A (Sx 1A), Synaptosome-associated protein of 25 kD (SNAP-25), and synaptotagmin, couple electrical excitation to evoked secretion. Two vicinal Cys residues, Cys 271 and Cys 272 in the Sx 1A transmembrane domain, are highly conserved and participate in modulating channel kinetics. Each of the Sx1A Cys mutants, differently modify the kinetics of Cav1.2, and neuronal Cav2.2 calcium channel.

**Methodology/Principle Findings:**

We examined the effects of various Sx1A Cys mutants and the syntaxin isoforms 2, 3, and 4 each of which lack vicinal Cys residues, on evoked secretion, monitoring capacitance transients in a functional release assay. Membrane capacitance in *Xenopus* oocytes co-expressing Cav1.2, Sx1A, SNAP-25 and synaptotagmin, which is Bot C- and Bot A-sensitive, was elicited by a double 500 ms depolarizing pulse to 0 mV. The evoked-release was obliterated when a single Cys Sx1A mutant or either one of the Sx isoforms were substituted for Sx 1A, demonstrating the essential role of vicinal Cys residues in the depolarization mediated process. Protein expression and confocal imaging established the level of the mutated proteins in the cell and their targeting to the plasma membrane.

**Conclusions/Significance:**

We propose a model whereby the two adjacent transmembranal Cys residues of Sx 1A, lash two calcium channels. Consistent with the necessity of a minimal fusion complex termed the excitosome, each Sx1A is in a complex with SNAP-25, Syt1, and the Ca^2+^ channel. A Hill coefficient >2 imply that at least three excitosome complexes are required for generating a secreting hetero-oligomer protein complex. This working model suggests that a fusion pore that opens during membrane depolarization could be lined by alternating transmembrane segments of Sx1A and VGCC. The functional coupling of distinct amino acids of Sx 1A with VGCC appears to be essential for depolarization-evoked secretion.

## Introduction

A physical and functional coupling of the VGCC with synaptic proteins provides a close apposition of the Ca^2+^ signal with the secretory machinery which is deemed crucial for the fast process of synaptic transmission [1]–[4]. It has been postulated, that a signal initiated by a conformational change during membrane depolarization at the pore of the channel, could trigger the fast secretion of “channel-associated vesicles” [5]–[8]. The idea that conformational changes could initiate secretion within microseconds is attractive because it might account for the rapid process of release that begins tens of microseconds after VGCC activation at the presynaptic release site [9]. Several members of the vesicle release machinery, including Sx 1A, SNAP-25, VAMP2/synaptobrevin, and synaptotagmin, interact with the cytosolic motifs of Cav1.2, and Cav1.3 (L-type), Cav2.2 (N-type), and Cav2.1 (P/Q-type) [10]–[19]. A functional interaction of Cav2.3 (R-type) with Sx 1A, SNAP-25, and synaptotagmin was also reported [20].

*In vitro* studies have shown physical binding of the cytosolic II-III domains of VGCC's, Cav2.2 (N_773–859_) [10], Cav1.2 (Lc_753–893_), and Cav2.2 (N_710–1080_) [2], [13], [14], [16] to Sx1A and other synaptic proteins. A specific site at the N-terminal of Sx 1A bound at N_773–859_, was shown to be responsible for Cav2.2 function [21].

Functional domain analysis revealed an additional site within the transmembrane domain (TMD) of Sx1A that could modulate Cav1.2 and Cav2.2 kinetics [21]. A double mutation at Sx 1A TMD, C271V/C272V, disrupted the Sx 1A inhibitory effect of Cav1.2 and Cav2.2 current amplitude [22], [23].

Different syntaxin isoforms sharing 23–84% identity have been described in various rat tissues, indicating distinct trafficking functions [24], [25]. Unlike Sx 1A, none of the TMD of these isoforms have vicinal cysteines [24]. The involvement of Sx isoforms in secretion differs in various cells. In adipocytes and muscle cells, Sx 4 was shown to participate in GLUT-4 exocytosis [26], [27]. Over expression of Sx 1A and Sx 3, but not of Sx 2 and 4, decreased insulin release in β-cells [28]. Sx 2, a Sx isoform whose TMD is less than 30% homologous to the Sx1A TMD, lowered Cav1.2 and Cav2.2 activation but had no effect on inward currents [22], [23].

We have examined the role played by the two highly conserved vicinal Cys residues in Sx1A TMD on evoked-secretion, using Sx 1A mutants, Sx isoforms, a vicinal Cys block by phenyl arsene oxide (PAO), and a truncated Sx 1A. Secretion was examined by monitoring membrane capacitance (Cm) in *Xenopus* oocytes co-expressing Cav1.2, Sx 1A, SNAP-25, and synaptotagmin, the excitosome proteins [13]. This functional reconstitution assay detected a depolarization-triggered release under voltage-clamp conditions with high precision and time resolution [29]. It is dependent on VGCC activation and on the presence of Sx1A, SNAP-25, and synaptotagmin. Release was triggered also in the absence of synaptotbrevin 2, suggesting the involvement of an endogenous tetanus toxin-insensitive synaptobrevin. Evoked-release was also sensitive to botulinum C1 and botulinum A [13] [29].

We show that a single point mutation at the TMD of Sx 1A, C271V (CC/VC) or C272V (CC/CV), disrupted voltage-evoked secretion, and propose a model to explain the requirement of the two TMD vicinal cysteines for supporting the secretion process. Our model suggests a simultaneous interaction of the two adjacent transmembranal Cys residues of Sx 1A with two VGCC molecules, and designates the VGCC as an essential member of the exocytotic-competent macromolecular cluster.

## Results

### Replacing cysteine residues of Sx 1A TMD abolished voltage-induced capacitance

**Figure 1:**
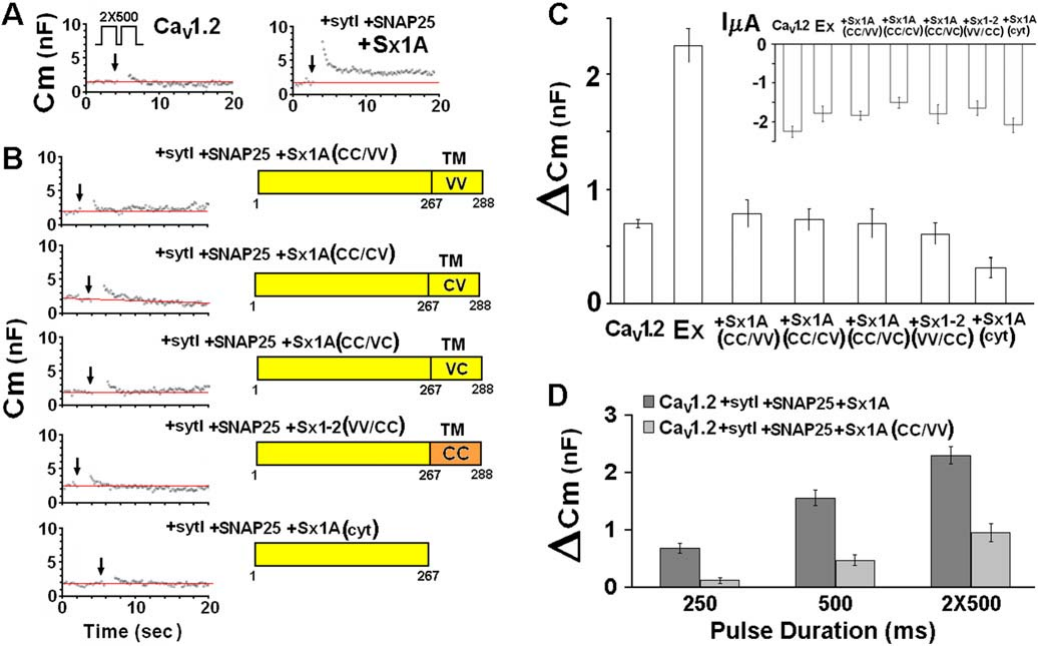
Mutation of Cys residues within the syntaxin 1A TMD disrupts depolarization-evoked capacitance transient. (*A*) *Upper* protocol, depolarizing voltage command consisting of depolarization from a holding potential of −80 mV to 0 mV for 2×500 ms, separated by 100 ms at −80 mV. Continuous monitoring of membrane capacitance of exemplary recordings, showing the effect of depolarization on membrane capacitance (C_m_) in an oocyte expressing, Lc-type Ca^2+^ channel (Cav1.2) subunits α_1_1.2, β2A, α_2_δ without (*left*) and with *SNARE's:* Sx1A 1A, SNAP-25 and synaptotagmin (*right*). The SNAREs and synaptotagmin were injected 24 hr after the injection of the channel subunits. (*B*). *Monitoring Cm in oocytes expressing different Sx 1A mutants- O*ocytes expressing heterologously Cav1.2 subunits (α_1_1.2, β2A, α_2_δ), SNAP-25, synaptotagmin I with either one of the Sx mutants: C271V/C272V, C272V, C271V, or V271C/V272C (Trus et al., 2001; Arien et al., 2003) or a truncated Sx 1A (1–167) (*C*) *Summary: effect of depolarization on C_m_.* Groups as in (*B*). *ΔC_m_*, depolarization-induced change of membrane capacitance; bars show mean±SEM (n = 13). *Inset,* The effect of depolarization on membrane current (InA mean±SEM, n = 13–18). (*D*) *Monitoring differences in Cm induced by different pulse duration via excitosome composed of Sx 1A and Sx CC/VV-* Capacitance induced by varying depolarizing pulses as indicated; bars show mean±SEM (n = 13–15).

We monitored whole cell membrane capacitance transients (Cm) induced by the activation of Cav1.2 subunits [α_1_1.2 (dN60-del1773), α_2_δ and β2A] without and with SNAP-25, Sx 1A and synaptotagmin (SytI) (excitosome complex) expressed in *Xenopus* oocytes, as previously described [29] (**Fig. 1A**). Two depolarizing pulses of 500 ms 100 ms apart, from a holding potential of −80 mV to 0 mV, according to the protocol in Fig. 1 [29]) were applied to oocytes co-injected with the corresponding proteins. The changes in Cm induced by Cav1.2 were 0.7±0.05 nF (n = 13) and by Cav1.2 with the synaptic proteins, 2.26±0.15 nF (n = 15) (**Fig. 1 A, C**). The Cm changes in *Xenopus* oocytes corresponds to the fusion of ∼10^5^–10^6^ cortical granules of 0.5–2 µm diameter [30] with an individual average membrane capacitance of ∼7–120 fF [29]. These values correspond to ∼1–3% of an oocyte's total membrane surface area.

The depolarization-induced capacitance change showed two distinct phases a fast transient component that was observed in oocytes expressing the Ca^2+^ channel alone (**Fig. 1A** ***left***) and a capacitance increase that was either stationary or slowly decaying, in oocytes expressing Cav1.2 in combination with syt1, SNAP-25, and Sx1A (**Fig. 1A** ***right***). Because of the nature of the transient increase in Cm, and to avoid possible current changes in Cm measurements as well as any contribution of a fast transient component to our signal, which could be due to the effect of ionic current, we determined Cm just after the current had returned to its previous basal level. These considerations although underestimating the extent of the exocytosis, provide more precise and convincing measurement of the change in capacitance [29]. It should be mentioned the Sx1A cRNA concentration used in the capacitance assay was within the linear phase of its activity on Cav1.2 current amplitude [22], [23].

Membrane-depolarization of oocytes expressing the multiprotein complex centered on Cav1.2 channels in which Sx 1A wt was replaced with either single or double Cys mutants, Sx1A C271V (CC/VC) or Sx1A C272V (CC/CV) or Sx1A C271V/C272V (CC/VV), showed a basal increase in membrane capacitance that was similar to those elicited by Cav1.2 expressed alone (0.71±0.05 nF; n = 13) (**Fig. 1B,C**).

### Sx1A/Sx 2 chimera failed to support voltage-induced secretion

To separate the role of the two vicinal Cys from the other amino acids of the Sx TMD, we studied Cav1.2 interactions using a Sx 1-2 chimera, derived from the Sx 1A cytosolic domain and Sx 2 TMD [22]

The chimeric molecule unlike Sx 1A *wt*, had no effect on current amplitude. When the chimera was further mutated by replacing two vicinal Val with two vicinal Cys at the TMD, it restored interaction with the channel [22]. However, when used in the release-assay, despite the presence of the two Cys residue, depolarization-evoked Cm changes corresponded to basal levels (**Fig. 1B**). These results indicate a likely contribution of other TMD amino acids to exocytosis required perhaps, for the lining of the fusion pore [31] or for the correct folding and organization of the excitosome into a competent secretory cluster (see below: **Fig. 1B,C**).

Next, we examined replacement of Sx 1A with a truncated Sx 1A (Cyt) mutant (amino acids 1–267; [15], [24]). As shown, depolarizing pulses evoked no change in membrane capacitance (**Fig. 1B,C**). Average Cm jumps monitored in groups of oocytes expressing excitosome complexes consisting of single, double, and cytosolic Sx1A mutants (above) are shown (**Fig. 1C**; n = 11–15).

Cav1.2 current amplitude was only marginally affected by substituting the various Sx1A mutants (**Fig. 1C** ***inset***). In the absence of any changes in Ca^2+^ influx, the large increase in Cm observed with wt Sx1A, indicate a better coupling and a more efficient assembly of the excitosome with Sx1A *wt*, rather than a result of a larger cation influx.

### Pulse duration influences depolarization-evoked secretion

The kinetics of release triggered by varying the length of the depolarizing pulses according to von Gersdorff [32], was recorded for excitosome complexes composed of Sx1A *wt* or Sx 1A (CC/VV) double mutant (**Fig. 1D**). Oocytes expressing the corresponding excitosome complexes were depolarized by pulse durations of 250 ms, 500 ms or 2×500 ms 100 ms apart. Unlike Sx 1A *wt*, the Sx1A (CC/VV) mutant showed capacitance transients of magnitudes similar to those induced by Cav1.2 expressed without synaptic proteins, indicating an ‘inactive’ secreting complex (**Fig. 1C, D**). We cannot rule out possible contribution to Cm that arises from an increase in intracellular divalent cations that promotes a Ca^2+^-dependent, SNARE independent process, similar to caffeine and ionomycin [33], [34]. Therefore, as voltage pulse duration increased and more channels were opened Cm was elevated in both complexes. Even under these conditions, of larger Ba^2+^ influx, the ineffectiveness of Sx1A(CC/VV) was apparent.

### Expression and targeting of Cav1.2 and syntaxin 1A mutants to the cell membrane

The obliteration of the capacitance response by substituting one of the Sx 1A mutants was substituted for Sx1A could result from difficulties in protein expression or targeting to the cell membrane, or both. Therefore, we prepared the fluorescent fusion proteins, RFP-Sx1A wt and RFP-Sx1A(CC/VV), that enabled us to evaluate protein expression and localization using confocal imaging.

**Figure 2:**
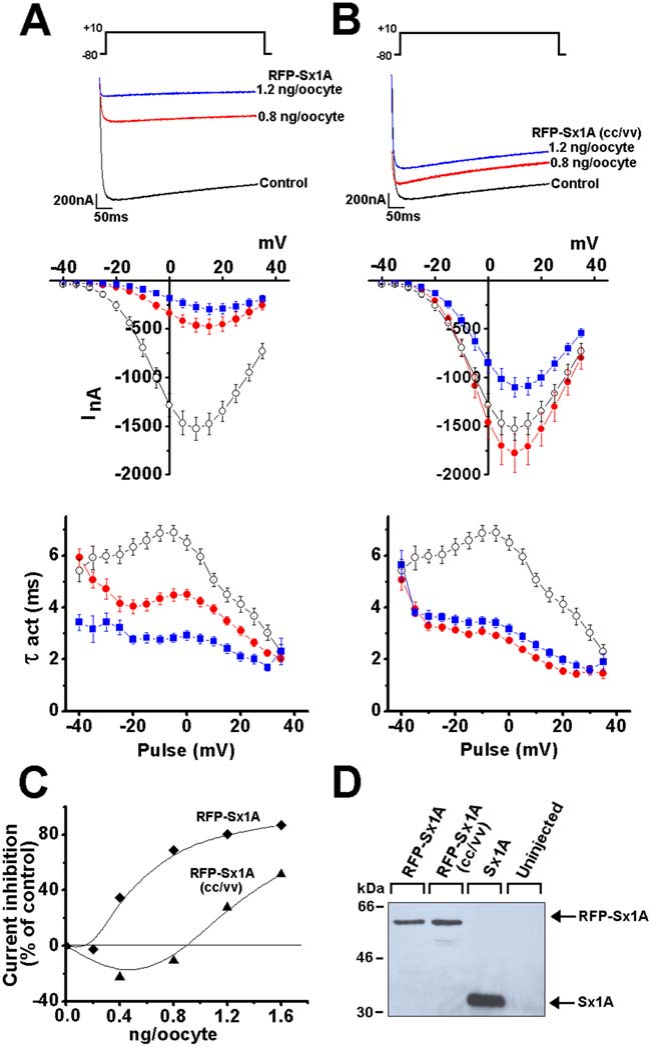
Expression and interaction of RFP-Sx1A and RFP-Sx1A(CC/VV) with Cav1.2. Superimposed current traces of GFP-α_1_1.2/β2A/α2/δ (5/5/5 ng/oocyte) co expressed with (A) RFP-Sx1A (0.8 ng/oocyte and 1.6 ng/oocyte) or (B) RFP-Sx1A(CC/VV) (0.8 and 1.6 ng/oocyte) in 10 mM Ba^2+^. Currents were elicited from a holding potential of –80 mV to +10 mV in response to 500 ms pulse (*upper panels*). Leak-subtracted peak current–voltage relations collected data from oocytes expressing GFP-α_1_1.2/β2A/α2/δ (5/5/5 ng/oocyte) without (-○-) and with RFP-Sx1A (0.8 ng/oocyte -•- and 1.6 ng/oocyte -▪-) (A) and RFP-Sx1A(CC/VV). Currents were elicited in response to 500 ms pulse from a holding potential of –80 mV to various test potentials at 5-sec intervals (B) (*middle*). Activation rate constants (τ, mean±SEM, n = 12) of currents generated in oocytes by GFP-α_1_1.2/β2A/α2/δ without (-○-) and with RFP-Sx1A (0.8 ng/oocyte -•- and 1.6 ng/oocyte -▪-) (A) and RFP-Sx1A(CC/VV) (B) (*lower*). The data points correspond to the mean±SEM of currents (n = 8-14) at each experimental point. Two-sample Student's t tests assuming unequal variance were applied, and P values <0.01 were obtained (C) Dose dependent of GFP-α_1_1.2/β2A/α2/δ current inhibition, plotted against increasing RFP-Sx1A and RFP-Sx1A(CC/VV) RNA concentration injected into oocytes. (D) Sx1A expression was tested in a western blot analysis of oocyte plasma membrane fraction co expressing GFP-α_1_1.2/β2A/α2/δ and 1.6 ng/oocyte RFP-Sx1A, 1.6 ng/oocyte RFP-Sx1A(CC/VV), 1.6 ng/oocyte Sx1A. Proteins were detected with anti-Sx1A antibodies.

Cav1.2 interaction with the two tagged-proteins was determined electrophysiologically by monitoring current kinetics in *Xenopus* oocytes injected with cRNA encoding the three channel subunits GFP-α1C/β2A/α2δ with RFP-Sx1A or RFP-Sx1A(CC/VV). Inward currents were elicited from a holding potential of −80 mV to various test potentials in response to 500 ms and recorded using the two-electrode voltage-clamp assay ([6], [14], [15] **Fig. 2**). Consistent with Sx 1A wt ([6], [15]) current amplitude elicited to +10 mV in oocytes expressing 0.8 or 1.2 ng/oocyte of RFP-Sx1A, was reduced as shown by superimposed current traces (**Fig. 2A** ***upper***). The Sx1A cRNA concentration used was within the linear phase of its activity on Cav1.2 current amplitude as previously determined [22], [23].

RFP-Sx1A(CC/VV) marginally affected current amplitude (**Fig. 2B** ***upper***), similar to the effect of the un-tagged Sx1A(CC/VV) ([22], [23]). Leak-subtracted peak currents from oocytes co expressing the three channel subunits and RFP-Sx 1A, or RFP-Sx 1A (CC/VV) were plotted as current-voltage relationship, showing current modulation at various test potentials (**Fig. 2A,B** ***middle***). The rate of activation, demonstrated by the time constant of activation (τact) was accelerated to the same extent by the two RFP-Sx1A proteins (**Fig. 2A,B** ***lower***) consistent with the untagged Sx1A [22], [23].

The difference in current modulation is depicted by a shift in RFP-Sx1A and RFP-Sx1A(CC/VV) concentration dependency (**Fig. 2C**). The absence of the Cys271 and Cys 272 interaction with the channel could be held responsible for the Sx 1A (CC/VV) inability to support release (see Fig. 1).

**Figure 3:**
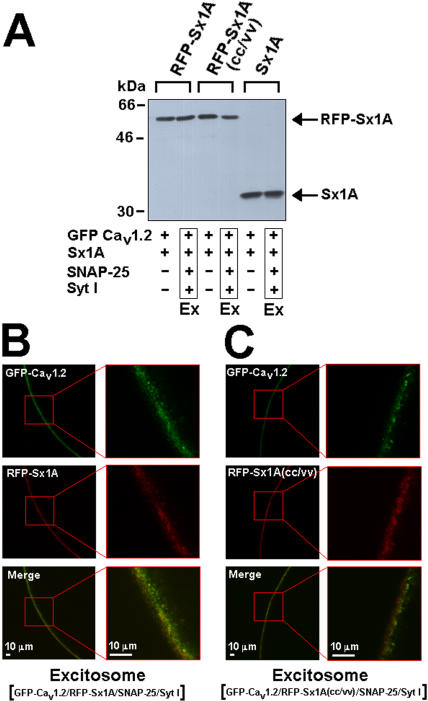
Expression and localization of Sx1A and Sx1A(CC/VV) on the cell membrane. (A) Western blot analysis of membrane fraction of oocyte expressing GFP-α_1_1.2/β2A/α2/δ (5/5/5 ng/oocyte) with either RFP-Sx1A (1.6 ng/oocyte) or RFP-Sx1A (CC/VV) (1.6 ng/oocyte) or Sx1A (1.6 ng/oocyte), with or without SNAP-25 (1.6 ng/oocyte) and Syt I (3.2 ng/oocyte) (Excitosome, Ex), using anti-Sx1Aa antibodies. (B) Oocytes were injected with cRNA mixture encoding the excitosome complex (GFP-Ca_v_1.2/RFP-Sx1A/SNAP-25/SytI) or (C) (GFP-Ca_v_1.2/RFP-Sx1A(CC/VV)/SNAP-25/SytI) and fluorescence was measured using confocal microscopy. GFP-Ca_v_1.2 fluorescence (*upper panel*) and RFP-Sx1A fluorescence (*middle panel*) were localized at the cell membrane and a merge of the showed co-localization of both proteins. The enlarged area is depicted at the right hand side. The experiment was repeated two times with 4 oocytes in each group.

After establishing that the RFP incorporation into Sx1A did not interfere with the Sx1A/Cav1.2 interaction, we measured RFP-Sx1A and RFP-Sx1A(CC/VV) levels in the cells. Oocytes co-expressing the channel subunits and RFP-Sx1A or RFP-Sx1A(CC/VV) were lysed and the proteins were separated on SDS-PAGE, blotted into nitrocellulose membrane and detected using specific Sx1A antibodies (**Fig. 2D**). Western blot analysis showed that the protein level of the tagged-proteins was lower than non tagged Sx1A wt, most likely due to the incorporated RFP-tag, but RFP Sx1A and RFP- Sx1A(CC/VV) were equally expressed, demonstrating that the mutation did not affect protein expression (**Fig. 2D**). Similarly, RFP- Sx1A and RFP-Sx1A(CC/VV) levels within the excitosome (Ex) were detected (**Fig. 3A**).

Targeting of the excitosome complexes Cav1.2, SNAP-25, Syt I Sx1A or Sx1A(CC/VV) to the cell membrane was detected by confocal fluorescence imaging of GFP-α_1_1.2 (**Fig. 3B,C** ***top***) and RFP-Sx1A or Sx1A(CC/VV) (**Fig. 3B,C** ***middle***). As anticipated from the functional α_1_1.2 interaction with Sx1A, both proteins were co localized and targeted to the cell membrane (**Fig. 3** ***lower*** *)*. Protein levels of RFP-Sx1A and RFP-Sx1A (CC/VV) (**Fig 3B,C** ***middle***) as well as current amplitudes were similar (**Fig. 1C** ***upper***) indicating a similar number of active channels at the cell membrane. Nevertheless, the fluorescence intensity of α_1_1.2/RFP-Sx1A (CC/VV) was smaller compared to α_1_1.2/RFP-Sx1A, suggesting a smaller number of clusters. Further studies using higher resolution systems are required to confirm putative changes in clusters size and distribution.

### PAO interferes with syntaxin 1A /VGCC interaction and decreases depolarization-induced secretion

**Figure 4:**
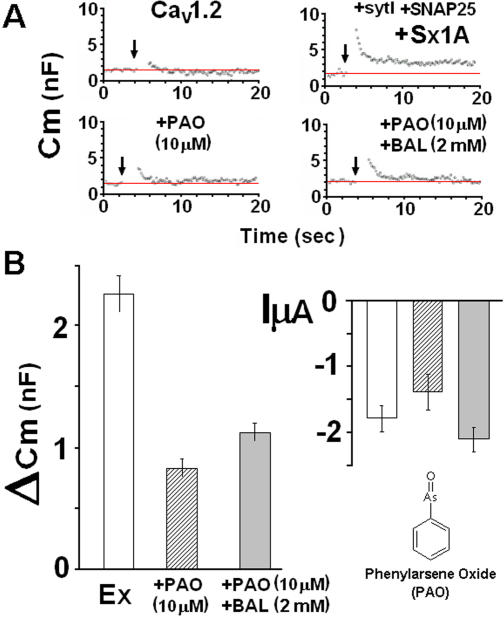
Phenyl arsene oxide abolished depolarization evoked ΔCm. (*A*) Continuous monitoring of membrane capacitance induced by a depolarizing voltage command from a holding potential of −80 mV to 0 mV of 2×500 ms, separated by 100 ms at −80 mV in an oocyte expressing, Cav1.2 subunits, α_1_1.2, β2A, α_2_δ without (upper *left*) and with *SNARE's:* Sx1A, SNAP-25 and SytI (*upper right*), and with either PAO (10 µM) (*lower left)* or PAO (10 µm) followed by 2 mM BAL (*lower right*). (*B*) Summary *of effect of* depolarization on C_m_. Groups as in the exemplary recordings shown in (*A*). *ΔC_m_*, depolarization-induced change of membrane capacitance; (*left*) bars show mean±SEM (n = 13) and the *Effect of depolarization on mean peak Ba^2+^ currents:* InA (mean±SEM, n = 11; *right* *).*

Phenyl arsene oxide (PAO), a thiol reagent that selectively reacts with two adjacent Cys residues to form a stable arsenic complex, interfered with the cross-talk of Sx 1A with the channel, most likely reacting with the vicinal Cys of Sx TMD and [23]. We tested the PAO effect on reconstituted secretion, applying 10 µM PAO directly into the recording bath (**Fig. 4A**). Membrane capacitance in oocytes expressing Cav1.2 along with the synaptic proteins, was monitored prior to PAO addition (Fig. 2A, *upper right*) and then, after 10s exposure to 10 µM PAO (**Fig. 4A**, ***lower left***). The ΔCm observed was similar to the change observed in oocytes expressing Cav1.2 without synaptic proteins (**Fig. 4A**, ***upper left***). An averaged ΔCm signal in 10 oocytes treated with PAO is shown in **Fig. 4B, C**. The oocytes were then exposed for 2.5 min to 2 mM 2,3-dimercaptopropanol (BAL), a reagent that reverses PAO reaction [35]. The external application of BAL to PAO-treated oocytes, resulted in a small but significant reversal of the PAO inhibitory effect (Fig. 2A, ***right lower*** and **Fig. 4B** ***left***). The partial effect of BAL might be due to irreversible changes at the oocyte membrane, which could have affected directly the capacitance measurement [23]. The high selectivity of PAO for vicinal cysteines provides additional evidence for the importance of the highly conserved Cys residues of the TMD of Sx 1A to depolarization-evoked capacitance. As shown in **Fig. 4B**, ***right***, oocytes treated with PAO, or PAO+BAL displayed statistically similar current amplitudes and could not account for the changes in Cm transients (see [29], [36]) (n = 11–12).

Although PAO's effect on secretion could be attributed also to its other activities in the cell, it had no direct effect on Cav1.2 amplitude; it acted rapidly (∼2 sec) to reverse the syntaxin effect and fully restored current amplitude [22]. The PAO results on secretion are consistent with those of Sx1A mutants, and support the possible involvement of two adjacent Cys in the release process.

### Syntaxin isoforms affect the kinetic properties of Cav1.2

**Figure 5:**
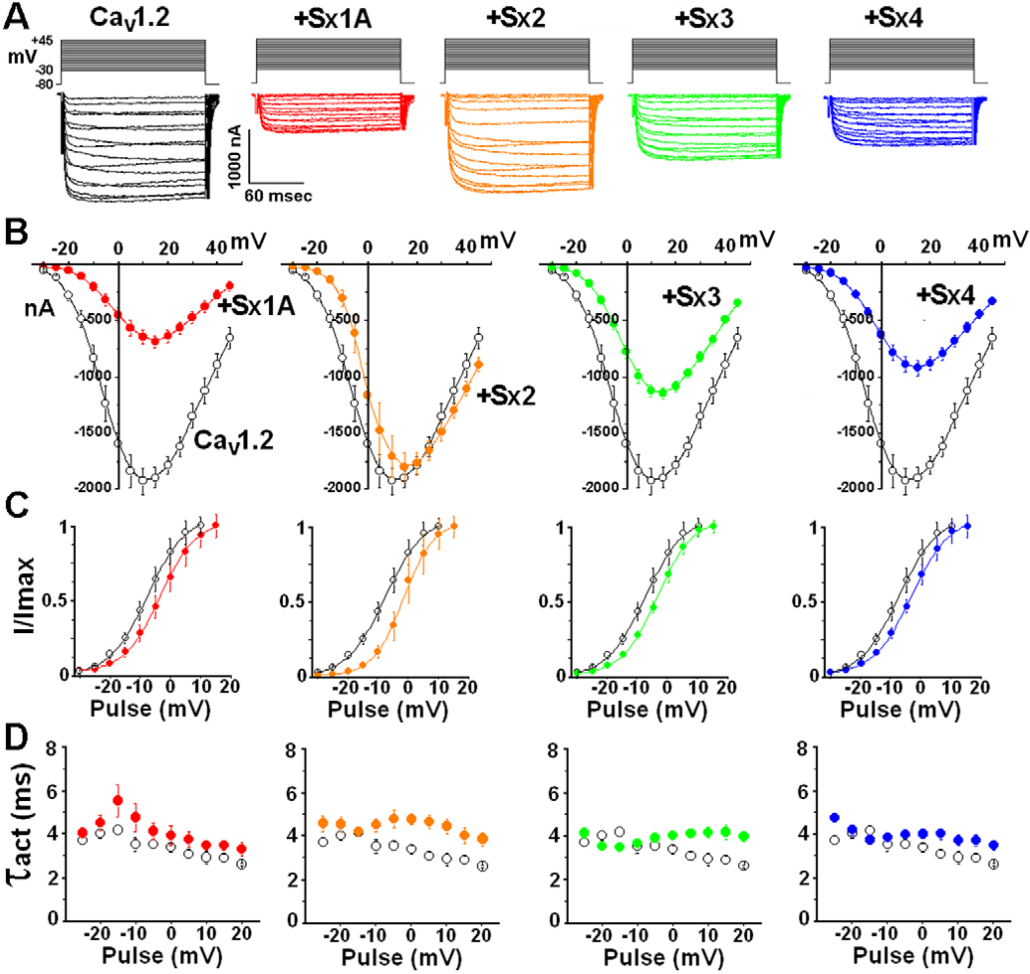
Modulation of Cav1.2 kinetics by syntaxin isoforms. Oocytes were injected with α_1_1.2 (2 ng/oocyte), β2A (5 ng/oocyte), α_2_δ (5 ng/oocyte) and 24 hr later, with either one of the syntaxin isoforms (2 ng/oocyte). (*A*) At day 6 after cRNA injection Ba^2+^ currents were elicited from a holding potential of –80 mV by voltage steps applied at 5-sec intervals to test potentials between –35 to +45 mV in response to 160 ms pulse duration. Representative traces of inward currents are shown. (*B*) Leak subtracted peak-current relationship: collected data form oocytes expressing Cav1.2 (o) and Cav1.2 with each one of the Sx isoforms (•). The data points correspond to the mean±SEM of currents (n = 8) at each experimental point. (*C*) The effect of syntaxin isoforms on I/Imax ratio. Peak current amplitudes normalized to maximum current (I/Imax) (data from B) are plotted against test potentials and displayed with a Boltzmann fit (mean±S.E.M; n = 8–10; more details in Experimental Procedures). (*D*) The averaged time constant of activation (τact mean±S.E, n = 11–13) are plotted against test pulses between −25 and +20 mV in the absence of (o) and in the presence (•) of Sx isoform as indicated.

To expand and further complement our insight of Sx1A role in depolarization evoked-release, we compared the ability of three Sx 1A isoforms, Sx 2, Sx 3, and Sx 4 to modulate Cav1.2 kinetics. Cav1.2 was expressed with each one of the Sx isoforms and inward currents were elicited in response to a 160 ms pulse, from a holding potential of −80 mV to various test potentials, according to the voltage protocol [6]; **Fig. 5A**). Superposition of current traces and current-voltage relationship of several oocytes are shown (**Fig. 5A,B**). Cav1.2 current amplitude was significantly decreased by Sx 1A, and to a smaller extent, by Sx 3 and Sx 4, but not by Sx 2 (**Fig. 5A**). All four isoforms showed a similar shift towards positive potentials as depicted in the I/Imax ratios (**Fig. 5C**). All Sx isoforms caused an increase in the time constant of activation (τact) at voltages above 0 mV, while Sx 1A increased τact also at negative potentials (**Fig. 5D**).

**Figure 6:**
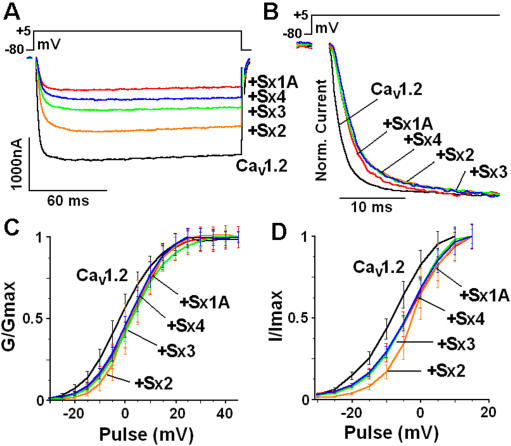
Comparison of syntaxin isoforms effects on the kinetics of Cav1.2. Cav1.2 subunits were co-injected with syntaxin isoforms (data from Figure 3). Ba^2+^ currents were elicited from a holding potential of –80 mV by a voltage step applied to +4 mV in response to 160 ms pulse duration. (*A*) Superposition of representative online leak-subtracted current traces measured with Sx isoforms as indicated with voltage protocols diagramed at the top. (*B*) The first 30 ms of the response is shown. The normalized traces show a shift of Cav1.2 activation by Sx isoforms. (*C*) Normalized conductance–voltage (G/Gmax) relationship obtained from (Fig. 3.B) displayed with a Boltzmann fit. The mid-point of activation (V1/2) and the Boltzmann slope (k) of Cav1.2 were: V1/2 = −7.6±0.2 mV, k = 6.3±0.3; with Sx 1A, V1/2 = −3.5±1.9 mV; k = 5.9±1; with Sx 2, V1/2 = −1.8±1.9 mV; k = 5±0.6±1; with Sx3, V1/2 = −3.7±0.9 mV, k = 5.9±0.5; and with Sx 4, V1/2 = −3.5±1.4 mV; k = 6.3±0.7. (*D*) Peak current amplitudes normalized to maximum current (I/Imax) (data from Fig. 3B) are plotted against test potentials and displayed with a Boltzmann fit. The data points correspond to the mean±S.E.M. (n = 10–12). Statistical significance was determined by Student's t-test.

For assessment of syntaxin isoform modulation of Cav1.2 kinetics, representative current traces of Cav1.2 co-expressed with each Sx isoform were superimposed. Currents were evoked by a voltage step from a holding potential of –80 mV to +5 mV in response to a 160 ms pulse (**Fig. 6**). A graded effect on the decrease in current amplitude was observed with Sx 3<Sx 4<Sx 1A. Sx 2 displayed no inhibitory effect (**Fig. 6A**; [22], [23]). All Sx isoforms shifted considerably the Cav1.2 normalized current, confirming functional interaction with Cav1.2 (**Fig. 6B**). The ratio of G/Gmax was shifted towards positive potentials by all Sx isoforms (**Fig. 6C**) and the largest shift of I/Imax was observed for Sx 2 (**Fig. 6D**). Consistent with previous reports [24]–[28], the functional modulation of the channel kinetics by the Sx isoforms similar to Sx 1A, shows a correct functional engagement with the channel.

### Sx 1A supports depolarization-induced capacitance increase

**Figure 7:**
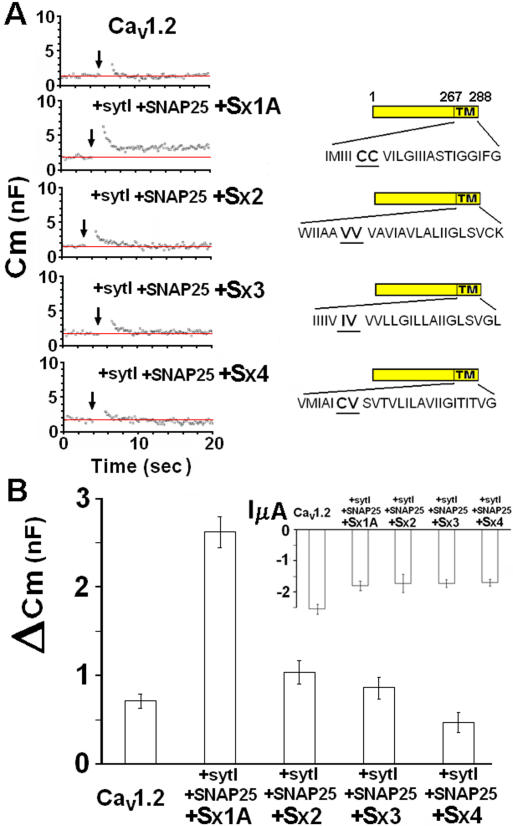
Assembly of the excitosome with syntaxin isoforms does not support depolarization-induced secretion. (*A*) Oocytes were injected with Cav1.2 subunits (as detailed in legend to Fig. 1) and 24 hr later with cRNA encoding SNAP-25, Syt 1 and either one of the syntaxin isoforms. Capacitance steps were elicited by two consecutive pulses of 500 ms, 100 ms apart as depicted in the protocol in Fig. 1D. Monitoring Cm in representative oocytes expressing heterologously Cav1.2 without and with SNAP-25, synaptotagmin and different syntaxin 1A isoforms. The amino acid sequence of Sx isoforms TMD are shown (*left)*. (*B*) Summary of the exemplary recordings shown in (A) of the Sx isoforms effect on Cm induced by membrane depolarization of Cav1.2 0.71±0.07 nF; n = 11; and with: Sx 1A, 2.65±0.17 nF (n = 20), Sx 2, 1.04±0.13 nF (n = 16), Sx 3, 0.86±0.12 nF (n = 12) and Sx 4, 0.48±0.11 (n = 12) (Groups as in A). *insert,* Average the corresponding peak currents amplitudes of VGCC expressed with SNAP-25, synaptotagmin and syntaxin isoforms, as indicated.

Given this framework, we next tested each of the syntaxin isoforms for supporting depolarization-evoked release. Oocytes expressing Cav1.2 without, and with Sx 1A, SNAP-25, and SytI (excitosome) were depolarized according to the protocol in Fig. 1, and Cm monitored (**Fig. 7A** *left*). Using the same depolarizing protocol, Cm was monitored in oocytes injected with a cRNA mixture of Cav1.2, SytI, SNAP-25 and one of the Sx isoforms, Sx 2, Sx 3 or Sx 4 (**Fig. 7A**). The amino acid sequences of the TM domains of Sx isoforms are indicated in **Fig. 7A**, ***right***. The averages of capacitance transients (ΔCm) were: for Sx 1A, 2.65±0.17 nF (n = 18), Sx 2, 1.04±0.13 nF (n = 16), Sx 3, 0.86±0.12 nF (n = 12) and Sx 4, 0.48±0.11 nF, while ΔCm of Cav1.2 alone was 0.7±0.1 nF (**Fig. 7B *left panel***). Thus, except for Sx 1A, none of the Sx isoforms supported depolarization-evoked release. Cav1.2 current amplitude recorded in the presence of the synaptic proteins was only slightly smaller, and not correlated to the calculated Cm (**Fig. 7B**, *inset)*.

## Discussion

We have used a functional reconstituted assay of secretion in *Xenopus* oocytes [29] as an approach to studying the crosstalk between Sx 1A and VGCC in exocytosis, and a means of examining the role of VGCC activation during the exocytotic events.

Whole cell membrane capacitance transients were monitored in oocytes expressing the recombinant excitosome complex consisting of Cav1.2, Sx 1A, SNAP-25, and SytI [13] or mixtures where Sx 1A was replaced by Sx1A TMD mutants, the Sx 1A/Sx 2 chimera, truncated Sx 1A, or different Sx isoforms.

### Sx 1A TMD mutants

We examined three Sx 1A TMD mutants: C271V (CC/CV), C272V (CC/VC) C271V/C272V (CC/VV) and a truncated cytosolic Sx1A (1–267) missing the TMD [15], [22].

Previously, it was shown that when a single Val residue was used to replace either one of the two highly conserved Cys 271 or Cys 272 residues of the Sx 1A TMD, modifications of Cav1.2 kinetics was similar to *wt* Sx 1A [22], [23]. A loss of current modulation was observed only when both vicinal Cys were mutated.

Despite the similar modulation of Cav1.2 kinetics by either one of the the single Cys mutants or wt Sx1A, we showed that neither CC/CV nor CC/VC supported depolarization-evoked release. These results reveal that both of the adjacent Cys residues are essential for promoting release.

### Sx 1A/Sx 2 chimera

Sx 2 has two vicinal Val residues, instead of two Cys at the corresponding position in the Sx 2 TMD. As previously shown, Sx 2 did not lower VGCC current amplitude, consistent with the need of two vicinal Cys residues for modulating inward current [24]. Hence, if the two Cys were solely responsible for decreasing VGCC current amplitudes, mutating Val residues to Cys at the Sx 1-2 chimera (constructed by cytosolic Sx1A and transmembrane domain of Sx 2) would restore inhibitory activity. Indeed, the mutated chimera, Sx1-2 (VV/CC), restored the predicted inhibitory activity [2], [22]. Despite this gain of activity, Sx1-2 (VV/CC) did not support release, indicating that in addition to Cys 271 and Cys 272, other amino acids at Sx 1A TMD play a role in the process of evoked release. These results reflect more stringent requirements for the release process, and are consistent with amperometry studies in PC 12 cells, where over-expression of only selective TMD Sx 1A mutants were reported to alter Ca^2+^-evoked release [31].

### Sx isoforms

The essential role of two vicinal Cys residues in depolarization evoked-release was further corroborated by the use of Sx isoforms, lacking the TMD vicinal Cys residues. Here we have demonstrated that none of the isoforms, supported evoked-release in the functional secretion assay. Unique sequences of both the cytosolic and transmembrane domains of Sx 1A appear to act in concert to support depolarization-evoked release. In further support of this, when Sx 1A is cleaved by botulinum C1, both evoked-release and modulation of VGCC currents [22] are lost [29], [37].

Previously, it was reported that in the retina, Sx 3 mediates presynaptic transmitter release from ribbon synapses, as opposed to the presynaptic Sx 2 and Sx 4 which are likely to mediate post-synaptic trafficking [38]. In an insulinoma cell line [28] and in pancreatic β cells, Sx 4 facilitates secretion of insulin over expression of Sx 1A and Sx 3 but not Sx 2 and Sx 4, strongly inhibited actions on insulin release [39]. In view of the data presented, it will be of interest to test whether Sx 2, Sx 3 and Sx 4 can support secretion perhaps, by interacting with different SNAP-25 and/or VAMP isoforms [40] .

### Proposed model

The most commonly accepted model for exocytosis is the SNAREs' model where the SNARE proteins (Sx.1A, SNAP-25 and synaptobrevin 2) are suggested to fuse with each other forcing the membranes into a close proximity to form a bilayer [41]. Initial reconstitution experiments using liposome containing recombinant SNAREs supported this model and led to the hypothesis that SNAREs by themselves are sufficient to account for the membrane fusion process [42]. However, un-physiologically high concentrations of SNAREs were required for the fusion and membranes breakage during fusion, have suggested that SNAREs alone are not sufficient to account for biological membrane fusion [43], [44]. These results raised the question whether the SNAREs just bring the membranes together or actually initiate fusion [45].

A more integral role of Sx 1A in the SNARE complex was implicated by its interaction with Munc-18 [46]–[48]. A tentative working model suggested that Munc18-1 promotes vesicle delivery most likely through the actin network [49] and then leads to vesicle docking [48]. According to this model, Sx1A/Munc18-1 dimer is formed and by unknown mechanism, transits to Munc18-1–SNARE complex. This complex was suggested to promote vesicle fusion by accelerating the fusion reaction [50].

Hence, in addition to the “leaky” type ‘SNARE's-only’ model, a more tightly controlled model was suggested which involves SNAREs, Rab, and SM proteins [45], [48], [51], [52]. See also [45], [48], [51], [52].

Our previous work has indicated that a minimal set of proteins assemble into a complex composed of the Ca^2+^ channel, tSNAREs and Syt1 [1], [5], [13], [14], [16], [23], [29], [53]. A functional assay was used that mimics several of the characteristics of exocytosis found in excitable cells; it is driven by membrane depolarization, sensitive to botulinum toxins (C and A), non-linear with divalent cation concentrations, and responds differently to various types of VGCC. In the functional secretion assay however, unlike in neuronal cells, synaptobrevin-2 contributed to an increase but was not absolutely necessary for secretion. This suggested the involvement of endogenous tetanus toxin-insensitive synaptobrevin (or cellubrevin) in the fusion process [29].

We propose here a working model that is based on a non-linear allosteric functional interactions of Sx1A with VGCC [29]. We have scrutinized the model in light of our results with the functional secretion assay that measures the fusion event. We assume that in our assay the vesicles in the oocytes are properly primed and associated with the VGCC [22], [23].

Consistent with our findings of the necessity of a minimal fusion complex, each Sx1A is in a complex with SNAP-25, Syt1, and the Ca^2+^ channel. The failure to support release when either of the two adjacent Cys residues in Sx 1A TMD are substituted by Val most likely results from structure alteration of Sx1A, that could affect the interaction and the allosteric regulation of the channel [23].

Based on a Hill coefficient of n_H_ = 2.02±0.2, indicating the non-linear dependency of Sx 1A concentration on current modulation [23], we propose that three (or more) Sx1A molecules assemble with equal number of Ca^2+^ channels to generate an exocytotic complex. The adjacent two Cys residues of Sx1A are proposed to play a role in the supra molecular assembly of channel molecules into a secreting competent complex [54]–[56].

**Figure 8:**
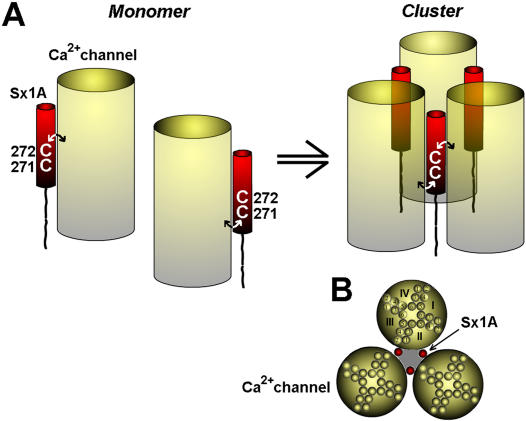
Assembly of VGCC Sx 1A, SNAP-25, and SytI, to generate a releasing complex; A Schematic model. The voltage -gated Ca^2+^ channel is schematically illustrated as a transmembrane barrel (*yellow*), and Sx 1A as a single transmembrane domain (*red*). (A) The Ca^2+^ channel, interact with Sx 1A transmembrane domain either via Cys 271 or Cys 272 residues, where one channel molecule interacts with one Sx1A (*left*). A simultaneous interaction of one Sx 1A molecule with two adjacent VGCC molecules via two vicinal Cys resides, lead to the Sx1A lashing two VGCC molecules, consequently, three Sx1A together with three VGCC molecules generate a secreting competent cluster (*right*). For simplicity, SNAP-25, and synaptotagmin were not inserted. (B) Top view of the cluster formed by three Sx1A and three Ca^2+^ channel molecules, clearly illustrates the fusion pore that traverses the plasma membrane to form a circle (*shaded area*).

Located far apart on a helical wheel model, we propose that one Cys residue could interact with one channel and the other, with a second channel (**Fig. 8A** ***left***). The two vicinal Cys at the Sx 1A TMD would enable one Sx 1A molecule to connect with two adjacent VGCC molecules. The association of three Sx1A with three channels would generate a cluster that mediates secretion (**Fig. 8A** *right*), and a top view (**Fig. 8B**). This would explain why a single Cys mutation could disrupt the assembly of the multiprotein complex thereby, obliterating secretion. The observation of an apparently lower number of clusters detected with confocal imaging of RFP-Sx1A(CC/VV) substituted for RFP-Sx 1A, would support this model. We believe that the hetero-oligomer complex generates a fusion pore (**Fig. 6B**; *shaded area)* that is lined by alternating transmembrane segments of Sx 1A and the calcium channel. Previously, a fusion pore formed by the circular arrangement of 5 to 8 Sx1A TMD segments was suggested [31], [57]

Our working model, which is based mainly on secretion of vesicles assembled with VGCC, does not preclude the participation of Munc18-1, Rab, or other proteins in the multiprotein complex. Further studies should clarify how and when does the Munc18-1/closed Sx1A dimer becomes a Munc18-1 trans SNARE complex, and whether Munc18-1/SNARE complex accelerates vesicle fusion via the multiprotein complex presented above.

*In summary*, unlike existing models of secretion, our model incorporates the VGCC as a pivotal member of the secretory complex. The close proximity of the channel to the exocytotic proteins allows for fast transmission of conformational changes from the channel to the release machinery. Secretion triggered by conformational change induced by cation bound at the channel selectivity filter prior to cation entry, implies a role of the VGCC as the Ca^2+^ sensor protein of secretion [7], [8]. Lastly, the channel as part of the exocytotic complex could be a constituent of the fusion pore, directly controlling the fusion process.

## Materials and Methods

*cDNA Constructs*—α11.2 (dN60-del1773; X15539 [GenBank]) and rat β 2a (m80545) were obtained from Dr. N. Qin and Dr. L. Birnbaumer (University of North Carolina); α 2/δ1 rabbit skeletal (M21948) [GenBank]) from A. Schwartz (University of Ohio). Sx isoforms, 2, 3, 4 were a kind gift of H. Y. Gaisano (Toronto Ontario, Canada). Sx point substitutions at residues 271 and 272 and Sx1A/Sx2 chimera, were previously described [22]. Anti syntaxin antibodies were the kind gift of M Takahashi (Tokyo, Japan).

### DNA constructs and RNA preparation

RFP-Syntaxin 1A (RFP-Sx1A) was prepared by insertion of the Eco47III-HindII RFP monomer fragment into EcoRV site of syntaxin 1A (#M95734). RFP-Syntaxin1A (CC/VV) was prepared by the quick-change method (Stratagene, LaJolla, CA) using RFP-Syx1A as a template with the primers- 5′ GGAAGAAGATCATGATCATCATTGTCGTTGTGATTCTGGGCATCATCATCGCC and 3′ CCTTCTTCTAGTACTAGTAGTAACAGCAACACTAAGACCCGTAGTAGTAGCGG. GFP-Ca_v_1.2 was a gift from Hagalili Yamit (unpublished data).

*In vitro* transcription kit was from Stratagene (La Jolla, CA). Phenyl arsene oxide and 2,3-dimercaptopropanol (British anti-Lewisite; BAL) from Sigma, Jerusalem.

### Heterologous protein expression in *Xenopus* oocytes

*cRNA Injection into Oocytes*—cRNAs were prepared using the T7/T3 Stratagene transcription kit, and the product was monitored by gel electrophoresis and optical density measurements.

Stage V and VI oocytes were surgically removed from female *Xenopus Laevis* and were injected with cRNA mixtures encoding Ca^2+^ channel subunits that were adjusted empirically to make the inward current lower than −4 µA. cRNA mixtures were injected into oocytes using a microdispenser (Drummond 510, Broomall, PA), in a final volume of 40 nl.

*Xenopus* oocytes were injected with a mixture of cRNAs encoding the Cav1.2 Ca^2+^ channel subunits α_1_1.2 (Lc-type (Δ1733); rabbit; 5 ng/oocyte) α2/δ (rabbit; 5 ng/oocyte) and β2A (rat; 10 ng/oocyte) and one day later either with water (for controls) or with a mixture of cRNAs encoding SNAP-25 (rat; 0.5 ng/oocyte, SytI (rat; 1.0 ng/oocyte) and Sx1A (0.5 ng/oocyte), or the equivalent amount of the corresponding isoform (as previously described [15]. Oocytes were kept in 18 °C until depolarization-induced exocytosis or inward currents were recorded after further incubation for 4 or 6 days.

### Capacitance monitoring in *Xenopus* oocytes

Membrane capacitance (Cm) was monitored in the two-electrode voltage-clamp configuration as published elsewhere [36]. Briefly, Cm was determined from the current response to a triangular, symmetrical voltage command of “Paired ramps” [45]. In a typical voltage stimulus, command voltage V_com_ increases by 40 mV within 20 ms, equivalent to a ramp slope of 2 V/s and *w*ith this slope, capacitance is obtained from the difference 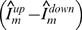 via simple division by 4:
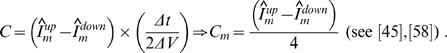
The up- and down-ramps (±20 mV in 20 ms each) elicit membrane currents that are the sum of resistive and a capacitive current component. Switching from up- to down-ramp reverses the sign of the capacitive component but not that of the resistive component. Thus, subtraction of the down-ramp current integral from the up-ramp current integral 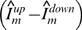 eliminates the resistive component; the resulting pure capacitive charge allows one to compute-together with the known amplitude of the voltage stimulus-membrane capacitance. Continuous monitoring is achieved by applying this stimulus repetitively at a high rate (up to 10/s; normally at 4/s). Comprehensive tests in an electrical cell model as well as in *Xenopus* oocytes have demonstrated high precision, accuracy, and robustness of this technique [36]. Starting from a holding potential of −80 mV, depolarizing stimuli were applied by clamping the cells to 0 mV for 2×500 ms, separated by 100 ms at −80 mV (unless stated otherwise). Capacitance was monitored before and after the stimulus, together with membrane potential (Vm) and current (Im).

### Confocal imaging

Single optical sections through the oocytes were acquired with an Olympus FV1000 (Olympus, Japan) equipped with a 40× oil objective (N.A. 1.3). Two excitation lasers were used sequentially: 488 nm for the GFP and 543 nm for RFP. Narrow-band emission filters 505–525 nm were used in the GFP channel and 560–620 nm in the RFP channel. Sequential scanning was performed with a resolution set to 512×512 pixels (0.621 µm/pixel), and single optical sections ∼0.5 µm thick were captured. Exposure time was 8 µsec/pixel. Protein expression and co-localization were analyzed using Image J software

### Membrane protein separation and Western analysis identification

Oocytes were homogenized (Kontes homogenizer) in buffer containing (mM): Tris–HCl 10 (pH 7.4), EDTA 1, sucrose 250 and 0.5% triton and a mixture of protease inhibitors: phenymethyl sulfonyl fluoride (PMSF), pepstatin A and luepeptin. Homogenates were then centrifuged (12,000×g, 10 min) to remove the yolk. Protein samples were quantified (Bradford reagent, BioRad, USA), using bovine serum albumin as a standard, then separated by 10% sodium dodecylsulphate–polymerase gel electrophoresis PAGE and detected using the electrochemiluminescent system using anti Sx1A.

### Electrophysiological assays

Whole-cell voltage clamp recordings were acquired from oocytes at day 5 or six after cRNA injection, as described previously [13]. To minimize Ca^2+ ^-activated Cl^−^ currents, oocytes were injected with BAPTA (final 5 mM) prior to recordings. Membrane currents were recorded by a two-electrode voltage clamp method using a DAGAN 8500 amplifier (Dagan). Bath solution contained (in mM): 5 Ba(OH)_2_, 50 *N*-methyl-D-glucamine, 1 KOH, 40 tetraethylammonium, 5 HEPES, titrated to pH 7.5 with methanesulfonic acid CH_3_SO_3_H.

Pulse duration for activation was 160 ms in 10 s intervals. Current traces were leak-subtracted on-line by the Clampex 8 software, and channel activation rates were analyzed by applying a mono-exponential fit (Axon instruments, Foster City, CA) to the current traces at the relevant ranges.
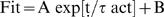
where A = current amplitude, τ = time constant, t = time to peak. Activation was determined from the beginning of the trace just after the capacitative transient to the peak-current region.

**Data Presentation and Statistical Analysis**-Peak current and time constant values analyzed by Clampfit 8 and transferred as ASCI file to an Excel worksheet (Microsoft Inc.). Data were averaged for each group of oocytes, and S.E. was determined. Data are presented as means±S.E. Statistical significance relative to the control group in each experiment was done by Student's t test by the Excel software. Statistical significance between multiple groups in each experiment was determined using one-way analysis of variance test using the Origin 6 software (Microcal). Final data was transferred to Origin 6 worksheet, plotted, and printed as final figures.
